# Complete Genome Sequence of a Wild-Type Isolate of Caulobacter vibrioides Strain CB2

**DOI:** 10.1128/MRA.01215-18

**Published:** 2018-11-01

**Authors:** Derrick C. Scott, LaTia Scott, Kiesha Wilson, Keshawn Ross, Damyen Ingram, Tajah Lewter, Jasmine Herring, LaTrice Linton, David Duncan, Anthea Aikins, Bert Ely

**Affiliations:** a Department of Biological Sciences, Delaware State University, Dover, Delaware, USA; b Department of Biological Sciences, University of South Carolina, Columbia, South Carolina, USA; Louisiana State University

## Abstract

The complete genome of Caulobacter vibrioides strain CB2 consists of a 4,123,726-bp chromosome, a GC content of 67.2%, and 3,896 coding DNA sequences. It has no rearrangements but numerous indels relative to the reference NA1000 genome. This will allow us to study the impact of horizontal gene transfer on caulobacter genomes.

## Abstract (precis)

The complete genome of Caulobacter vibrioides strain CB2 consists of a 4,123,726-bp chromosome, a GC content of 67.2%, and 3,896 coding DNA sequences. It has no rearrangements but numerous indels relative to the reference NA1000 genome.

## ANNOUNCEMENT

Caulobacters are Gram-negative bacteria which produce stalks at one end of the cell. The bacterium differentiates into two cell types and divides asymmetrically at each cell cycle. Caulobacter crescentus strain CB15 has a well-developed system of genetics (1, 2) and was the first caulobacter to be fully sequenced (3). It was developed into a single-celled model system to study cellular differentiation, asymmetric division, and cell cycle progression (4, 5). Studies of this obligatory differentiation during the cell cycle made caulobacter the dominant prokaryotic model system for studying the mechanisms of cell cycle control and cellular differentiation. Today, however, caulobacters have attracted interest because it was discovered that even closely related strains of caulobacters exhibit extremely high levels of genome rearrangements (6, 7). To discover the molecular mechanism behind this “genome scrambling,” more genomes are needed for genomic comparisons. Here, we report the full sequence of the genomic material of an additional Caulobacter vibrioides wild-type strain, CB2, sampled from tap water in California (8).

The Caulobacter vibrioides strain CB2 was grown in peptone yeast extract for 48 h at 30°C as previously described (9). Total genomic DNA was extracted with a Qiagen DNeasy tissue kit following the manufacturer’s protocol. Previous studies have shown that high-GC-content genomes can be challenging to sequence with short-read technology (10). As such, the total genome was sequenced with a PacBio RS II sequencer at the Delaware Bioinformatics Institute. The DNA library was prepared for sequencing with the PacBio blunt-end ligation protocol. The subsequent raw reads were assembled with the Hierarchical Genome Assembly Process (HGAP3) (11) in SMRT Portal with the default *de novo* parameters. The computational requirements needed for the analysis were leveraged through Amazon Machine Image (AMI) EC2 with the smrtanalysis-2.3.0-ami-20fb4848 image. The number of raw assembly reads was 45,003 with a *N*_50_ read length of 23,435 bp. Total coverage was 125×. All pipelines began with adapter removal and subread filtering with a final contig polish done by Quiver, the last step in the HGAP3 assembly process. The sequence was annotated with the Rapid Annotation using Subsystem Technology (RAST) server (http://rast.nmpdr.org) and the NCBI Prokaryotic Genome Annotation Pipeline (12–14) and then visualized in Artemis (release 16.0.0) (15). The CB2 genome aligns well with the circular Caulobacter NA1000 genome. The ends of the contig we obtained extensively duplicated each other, indicating circularity. The complete genome of Caulobacter vibrioides CB2 is 4,123,726 bp, with a GC content of 67.2%. There are 3,896 predicted coding sequences (CDSs), 52 tRNAs, and 2 rRNA operons.

Caulobacter vibrioides CB2 has no inversions, but it has numerous insertions and deletions compared to the closely related NA1000 strain. The absence of rearrangements indicates that this well-documented phenomenon (6, 7), occurs relatively rarely in these two compared genomes. Further studies will be able to shed light on the horizontal gene transfer that happens much more frequently among caulobacters.

### Data availability.

The complete genome sequence of Caulobacter vibrioides CB2 has been deposited in GenBank under the accession number CP023313. The raw reads are also available under SRA accession number SRX4603198.
